# The UK GenScan study - population-based imaging genetics research using 3D Cardiac Magnetic Resonance

**DOI:** 10.1186/1532-429X-15-S1-E2

**Published:** 2013-01-30

**Authors:** Antonio de Marvao, Tim Dawes, Niall G Keenan, Wenjia Bai, Wenzhe Shi, Giuliana Durighel, Tamara Diamond, Laura Monje-Garcia, Stuart A Cook, Declan P O'Regan

**Affiliations:** 1 Robert Steiner MRI Unit, MRC Clinical Sciences Centre, Imperial College London, London, UK; 2 Department of Cardiology, Imperial College Healthcare NHS Trust, London, UK; 3 Department of Computing, Imperial College London, London, UK

## Background

Cardiac structure and function results from complex interactions between genes, molecular regulators and environmental factors. Until recently, imaging-genetics studies had limited power as standard methods of cardiac phenotyping rely on global indices of mass and function, providing only macroscopic descriptors. Manual analysis of large cohorts is time consuming and subject to inter-observer variability.

Current cardiac magnetic resonance (CMR) cine imaging techniques are limited by poor through-plane resolution. Here we present a high resolution 3D left ventricular atlas with automated segmentation.

## Methods

Subjects: healthy volunteers (19), patients with dilated cardiomyopathy (DCM) (5) and hypertrophic cardiomyopathy (HCM) (5) from the Genetic Studies of the Heart and Circulation (GenScan) study. Ten volunteers had two studies to assess repeatability. A further five healthy volunteers were imaged to create the atlas.

Subjects underwent CMR using a 1.5T Philips Achieva system with a 32 element cardiac phased-array coil. Short axis SSFP images were acquired: 2D: voxel size 2.0 x 2.2 x 8.0 mm, 12 sections, two sections per breath-hold, slice thickness 8 mm with 2 mm gap, 30 cardiac phases. 3D: single breath-hold 3D b-SSFP volumes: voxel size 2 x 2 x 4 mm (reconstructed to 2 x 2 x 2 mm), 48 sections, 20 cardiac phases, SENSE factor 4.

**Figure 1:**
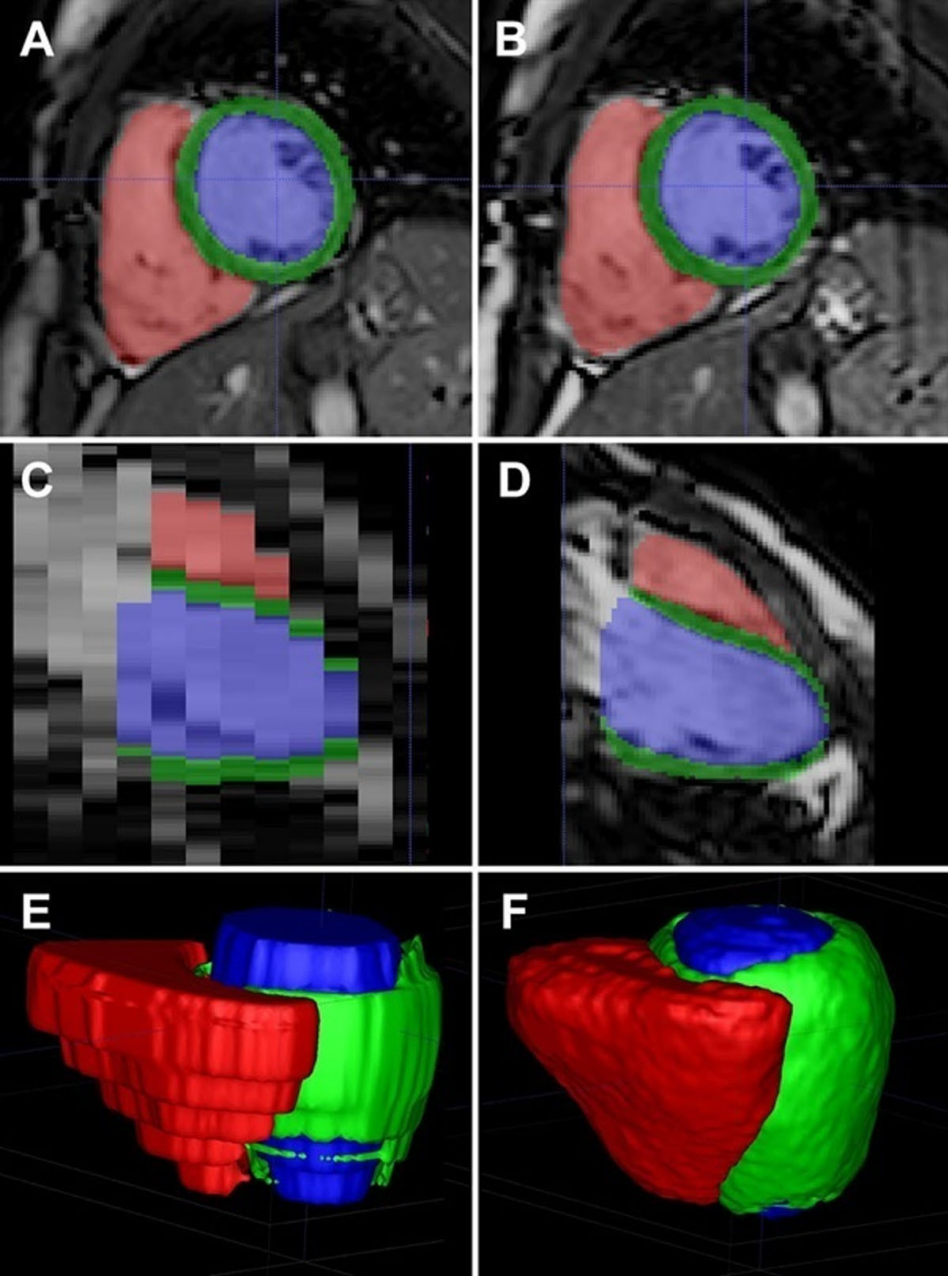
Three dimensional cine imaging and segmentation. Left column (A, C, E) show conventional 2D imaging with poor through-plane resolution. Right column (B, D, F) shows the high resolution segmentation that is possible using 3D single breath-hold imaging.

The 3D cine atlas was developed using ITKsnap software to manually label each voxel. Novel images were then segmented automatically using multi-atlas simultaneous segmentation and registration (Figure 1).

**Figure 2:**
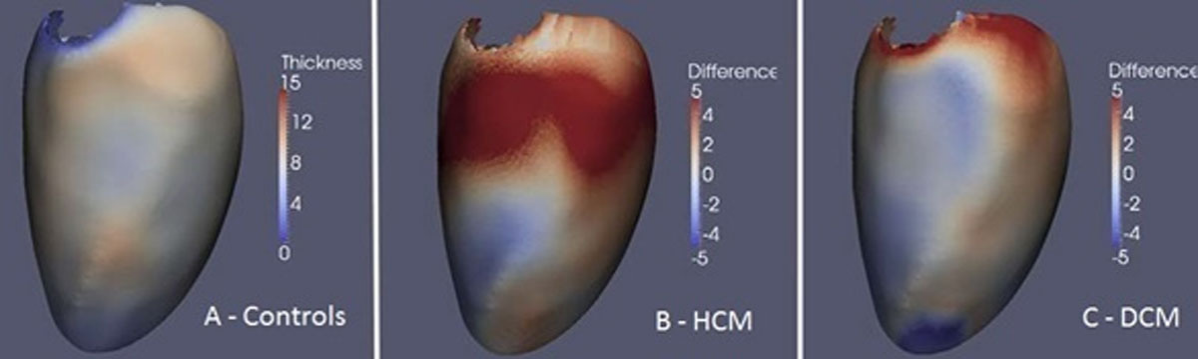
Average aortic wall thickness increase in ten years by age categories (in the baseline age).

Wall thickness was determined as the distance between each point on the epicardial surface and its closest counterpart on the endocardial surface. To assess accuracy conventional manual volumetry of the 3D images was made using Philips Extended Workspace. The segmentations of the LV were co-registered enabling 3D point-by-point comparisons of wall thickness between the three cohorts using parametric tests (Figure 2).

## Results

2D manual volumes (geometric means and 95% CIs) were similar to 3D automated volumes. LV end-diastolic volumes - 154mLs (141-169mLs) vs 156mLs (143-169mLs). Differences not statistically significant (one sample t-test, t=0.681, p=0.50). Mean automated volumetry (expressed as percentage of the whole) was 1% greater than 2D manual volumetry.

## Conclusions

High spatial resolution 3D cine imaging provides accurate assessment of LV volumes and mass and enables single breath-hold cardiac phenotyping. Co-registration permits pooling of data from large cohorts and is effective for voxel-wise statistical comparisons of wall thickness. This technique will be used for mapping the effect of genetic variants on regional function, morphology and strain in a high-throughput population based study.

## Funding

Supported by the Medical Research Council, Imperial College Biomedical Research Centre and the British Heart Foundation.

